# Efficient material flow in mixed model assembly lines

**DOI:** 10.1186/2193-1801-2-415

**Published:** 2013-08-28

**Authors:** Mohammed Alnahhal, Bernd Noche

**Affiliations:** Transport Systems and Logistics Engineering Institute, Mechanical Engineering Department, Duisburg-Essen University, Duisburg, 47058 Germany

**Keywords:** Material flow, Assembly line, Milk run, System variability

## Abstract

In this study, material flow from decentralized supermarkets to stations in mixed model assembly lines using tow (tugger) trains is investigated. Train routing, scheduling, and loading problems are investigated in parallel to minimize the number of trains, variability in loading and in routes lengths, and line-side inventory holding costs. The general framework for solving these problems in parallel contains analytical equations, Dynamic Programming (DP), and Mixed Integer Programming (MIP). Matlab in conjunction with LP-solve software was used to formulate the problem. An example was presented to explain the idea. Results which were obtained in very short CPU time showed the effect of using time buffer among routes on the feasible space and on the optimal solution. Results also showed the effect of the objective, concerning reducing the variability in loading, on the results of routing, scheduling, and loading. Moreover, results showed the importance of considering the maximum line-side inventory beside the capacity of the train in the same time in finding the optimal solution.

## Introduction

One of the most important types of waste in any manufacturing facility is material handling, especially in the case if there are a lot of different product models. If these product models are assembled on the same production line with very low setup time, Mixed Model Assembly Lines (MMAL) are used. This environment requires very variable feeding of parts at different stations where the number of parts required by each station in a short period is relatively low compared to the pallet size supplied by traditional transporters such as forklifts (Baudin 2004). Usually areas beside stations are scarce and not enough to hold a lot of inventory. Another strategy in which assembly workers can get parts in small bins very close to them is milk run system in which a group of stations is fed by the same tugger or tow train in the same route where each station gets its demand of parts for a certain period (Droste & Deuse 2011).

Sometimes, the feeding process using tugger trains is based on using Kanban system to “tell” the material handler (train driver) how many bins he should deliver in each route he makes at each station he visits (Ciemnoczolowski & Bozer 2013). However in other cases, the exact demand during the shift is exactly known before the beginning of the shift based on a predetermined sequence of product models where each model has a certain demand of parts at each station on which it is assembled (Golz et al. 2011). In this study the second case is assumed.

Usually, milk run train makes its routes from a central warehouse to different stations. However, sometimes assembly lines are very long, and the distance from the central warehouse to some of the stations is too long. In this case, another strategy is used in which there are scattered inventory areas which are used to feed the stations near them. Usually in the practice, every 20 to 30 stations are supplied by the same decentralized inventory called *Supermarket* (Battini et al. 2010; Emde & Boysen 2012a; Emde et al. 2012; Emde & Boysen 2012b).

Managing milk run trains offers a challenge for researchers because the variability in the system is usually high where in some times the loaded quantity by the train is near its full capacity while in other times the loaded quantity is low. Another factor is considering the line-side inventory where the area beside stations must not be crowded by a lot of bins which hinder other activities and increase the holding costs, and this contradicts with the principle of Just in Time (JIT) for which milk run system is designed.

In this study, the feeding process using milk run tugger train from decentralized supermarkets to MMAL stations will be studied. The problems of scheduling, routing, and loading of tuggers will be examined. Minimizing inventory costs, reducing the variability in the system in loading and in routes lengths, and reducing the resources needed (material handlers) will be the objective of the study. An example will be presented to explain the methodology. The main concentration of the methodology of this study, beside the analytical equations, is to utilize, organize, and modify the existing models found in the literature in a systematic way to achieve the objectives of the study.

The following sections will be organized as follows: Section “Literature review” presents the literature review concerning the decision problems regarding material flow in MMAL in the case of using decentralized supermarket system. Section “Methodology and example” presents the methodology consisting of the major steps, and presents an example. Section “Results and analysis” presents the results and the analysis of results. Finally Section “Conclusion” will present conclusion and recommendation for future research.

## Literature review

In a study by (Choi & Lee 2002), the feeding system was classified to be dynamic and static. In static part feeding system, the hourly consumption rate of parts is determined every day morning, and usually it does not change during the day. The dynamic part feeding system estimates the parts consumption amounts dynamically considering the actual production progress and directs the feeding orders dynamically to feeders. In our study, we strict ourselves to the first type of feeding. A study by (Caputo & Pelagagge 2011) investigated three feeding polices namely, kitting, just in time kanban-based continuous supply and line storage. However our study deals with a fourth policy, namely in-plant milk run which combines several advantages of the above three polices and gets rid of most of their disadvantages such as extra work of kit preparation and large inventory space requirements.

The planning and control of in-house logistics concept involves several interrelated decision problems such as (Emde & Boysen 2012a; Emde et al. 2012; Emde & Boysen 2012b):

i. Decide on the number and location of decentralized supermarkets.
ii. Determine the number of tow trains per supermarket and assign line segments to them.
iii. Determine each tow train’s fixed delivery schedule.
iv. Decide on the bins to be loaded per tour of a tow train.

Before investigating the first decision (i) in the above list, an important problem in the system is storage centralization/decentralization decision where the management has to decide if a central warehouse or decentralized inventory system will be used. For example, (Battini et al. 2010) considered investigating this decision where they studied the usage of supermarkets. Sometimes supermarkets are used to perform other tasks, however in this study we will only consider supermarkets as scattered decentralized storage areas serving as intermediate store to feed nearby stations. Some advantages of that is fast and frequent delivery of parts and freight consolidation by being supplied by industrial trucks. However, supermarkets consume space on the factory floor, which is scant and expensive (Emde & Boysen 2012b). For this decision also, a cost model was presented in a study by Sargent et al. (1995) where the model can be used to determine if further consideration should be given to decentralized storage in a facility currently utilizing centralized storage. The cost model examines the trade-off between the savings in material handling flow costs due to moving from centralized to decentralized storage and the additional costs associated with implementing and utilizing decentralized storage for a designated period of time. In a study by Battini et al. (2009), the level of inventory centralization and decentralization was investigated. According to the same study, different kinds of decentralization strategies can be studied, like stocking directly in the assembly station, stocking next to one station, or one stocking area for all assembly stations on the line. The main factors to determine which system to use are demand frequency for each component, average demand rate, average cost, size of the parts, available area next to the assembly stations, and batch size of components. The degree of inventory centralization/ decentralization in our study was predetermined where we have several supermarkets feeding every assembly line. So this stage is assumed to be input to the study. Satoglu et al. (Satoglu & Sahin 2012) evaluated the conversion from central storage to decentralized storages in cellular manufacturing environments using activity-based costing. Furthermore, (Alizon et al. 2009) investigated the flow of materials from several internal warehouses to supply the same assembly workshop. Moreover, in a study by (Hanson & Finnsgård 2012), a case study was presented in which the material feeding was organized by using three drop zones which were close to three assembly lines. These drop zones get the material from the AS/RS system. Then materials were transported from the drop zones to the different stations in the assembly lines.

The second (ii), third (iii), and fourth (iv) decisions investigate scheduling, routing, and loading problems which are the main focus of this study. Routing problem has special conditions in the system we study since the routing is only about determining the part of the assembly line that will be served by the same train. This is because in decentralized supermarket system, we have several small inventories scattered along the assembly line, and each one of these supermarkets can have several trains. So every train will supply a small segment of the long assembly line. So, traditional routing problems are not applied in this case (Emde et al. 2012). The scheduling problem investigated in this study is also special where we assume fixed time periods for each rout of the train. So the main two decisions here are to determine the length of this service period and also the starting time of the first route.

In the literature surveyed, the routing and scheduling were related to each other. Some studies assumed that routing is input and they investigated the scheduling problem. On the other hand, some studies assumed that the periods are the input, and they investigated the routing problem. However, other studies such as (Emde & Boysen 2012a) studied the routing and scheduling together. According to (Kilic et al. 2012), the periods in train routes scheduling can be fixed or variable. Emde & Boysen (2012a) showed that the variable periods are optimal for reducing inventory costs. However and from the point of view of lean manufacturing, it is better to standardize the system by fixing the periods. Some studies even considered fixed periods as a must to call the system as milkrun such as (Bozer and Ciemnoczolowski 2013). Moreover, (Hanson and Finnsgård 2012) presented a real case study in which the time period of the tugger train was constant. Satoglu and Sahin 2012 developed a mathematical model and a heuristic approach where the routes are constructed and the service period is determined for the design of an internal milk run material supply system. The service period was also investigated in other studies such as Domingo et al. (2007) and Álvarez et al. (2009).

Golz et al. 2011 studied the routing, scheduling, and loading problems for centralized supermarket systems to decrease the number of tow trains. In the area of decentralized supermarket system, little research was performed on the problems in the above list. Battini et al. 2010 and Emde and Boysen 2012b studied the problems of determining the number and locations of supermarkets. Emde and Boysen 2012a investigated the scheduling and routing problems together in parallel to minimize the line-side inventory holding costs. Assuming given routes as input, (Emde et al. 2012) investigated the loading problem to minimize the holding costs of line-side inventory in the case that there are bottleneck periods (routes) in which the demand of the stations served by the same train exceeds the capacity of the train. In this case, some bins must be delivered in previous non-bottleneck routes. In this study “early loading” will be used to define this case. Early loading must be controlled in a good way to minimize inventory holding costs. In a study by (Faccio et al. 2013), the fleet size (which is part of routing problem) and kanban number were investigated in static and dynamic steps of analysis. However, that study assumes using one supermarket feeding a group of assembly lines. In our study, we assume that the supermarket feeds only a group of stations in the same assembly line.

According to the best of the knowledge of the authors, there is no any previous work that combined routing, scheduling and loading problems and solved them in parallel in the same model. However, these problems are interrelated. So this study fills in that gap by investigating all the problems together in parallel, and minimizes inventory costs, variability in the system, and the number of trains. The biggest advantage of investigating the three problems together comes from the possibility of adding the objective of reducing the variability in loading. Without combining the three problems together, this objective cannot be achieved. Another advantage is to further decrease the total inventory holding costs especially in the case of using *early loading*. In previous studies, the maximum possible decrease in inventory holding costs is limited by the predetermined routing, which was considered as input and cannot be changed.

Moreover, the study investigates finding the optimal service period of the train in a different way in which this period is fixed, and can be determined analytically taking into consideration constrictions such as maximum line-side inventory, train capacity, and traveling time plus loading and unloading times. Doing so is important to simplify the system especially for the material handler.

## Methodology and example

The same assumption about the deterministic nature of demand of parts and assembly times which are found in (Emde and Boysen 2012a) will be adopted also in this study. To explain the methodology, an example is shown where there are 20 stations supplied by parts using tugger trains. The 20 stations are assumed to assemble only 4 types of product models (1, 2, 3, and 4). Table 1 shows the parts needed by each model on each station. It is assumed here that each station needs the same types of parts for all the four models. However, the parts are different from a station to another.

**Table 1:** **The needed number of parts required to assemble each model at each station**

Stations	Model				Stations	Model			
	1	2	3	4		1	2	3	4
1	2		1		11	1		3	1
2			2	1	12	1		1	
3	1	2			13		1		1
4	1	3	1		14	1	2		2
5	1			3	15		1		1
6	1		1		16		2		
7		1		1	17	1			1
8	1	2			18	1	3		
9	3		1	1	19		1		2
10		1		1	20	1	2		1

For simplicity, the sequence of assembling the four models was chosen to be 1 → 2 → 3 → 4. However it can be any other sequence. The sequence is assumed to be an input for the study and finding another sequence will not be investigated. In this study and as in the study by (Emde and Boysen 2012a), we will use the cycle time, which is the time required to assemble one product model at any station, as the time unit.

In the first cycle of work, the first model is assembled on the first machine consuming two parts as it is obvious in Table 2a which shows it in the first 10 cycles in the shift and for the first 9 stations. In the second cycle, the first model is not assembled on the second machine since it does not require any part. Also in the same cycle the second model is not assembled on the first machine. In the third cycle, the third model is assembled on the first machine consuming only one part. In the same third cycle, the first model is assembled on the third machine consuming one part. This mechanism is followed for all the work cycles during the shift.

**Table 2:** **The needed quantity of materials required at each cycle at each station**

Station	Cycle										Station	Cycle									
	1	2	3	4	5	6	7	8	9	10		1	2	3	4	5	6	7	8	9	10
1	2		1		2		1		2		1	1						1			
2				2	1			2	1		2				1					1	
3			1	2			1	2			3			1					1		
4				1	3	1		1	3	1	4				1				1		
5					1			3	1		5					1					
6						1		1		1	6						1				
7								1		1	7								1		
8								1	2		8								1		
9									3		9									1	
a. Demand in parts unit											b. Demand in bins unit										

After that and assuming that the capacity of the bins transported by the train is always 5 parts for all the types of parts, the demand of bins is calculated as in Table 2b for the first 10 cycles and for the first 9 stations. For example at the first station, we need at the first cycle two parts, so we need a bin at this cycle. The bin must come one cycle earlier, which is cycle 0. The three remaining parts in the bin can satisfy the demand for the third cycle and also for the fifth cycle. However in the seventh cycle, we need a new bin. This way is followed for all the cycles and stations.

We assume in this study that the tugger is capable of delivering the needed bins to a station after feeding the previous one in duration of one cycle. So, for example, for a certain time period, the tugger, after delivering all the needed bins to the first station, can deliver the other needed bins to the second station in the same period in duration of one cycle. However, this time is variable and depends on the number of delivered bins. Because of that, the decision maker may consider using three different types of buffers: safety stock, time buffer among routes, and line-side empty inventory buffer. So if the needed bins arrive too late, the safety stock is useful to replenish the stations until the next arrival of the train. Moreover, for this delay not to affect the next routes of the train, the time buffer among routes is useful in this case. However, time buffer should not be too long because this will decrease the feasible solution space as will be explained later. On the other hand, if the arrival of bins is too early, empty space in the line-side inventory can be useful.

### Study procedures and objectives

**Figure 1:**
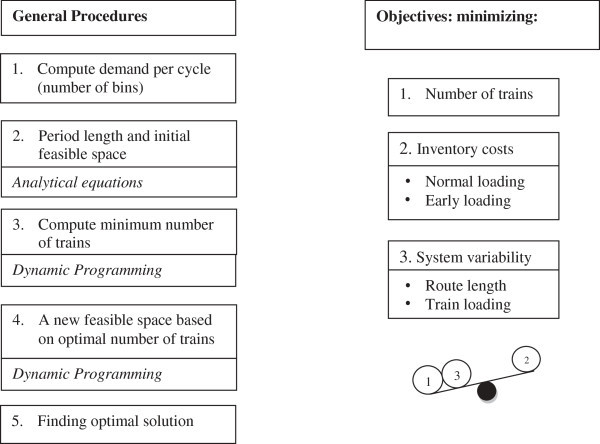
**Study major steps and objectives.**

**Figure 2:**
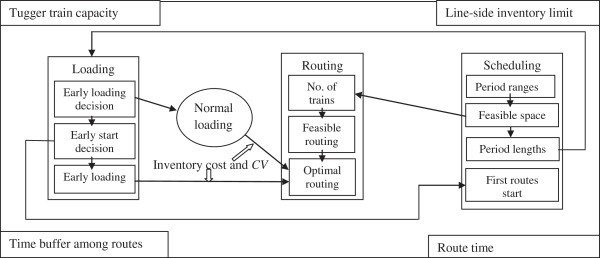
**Study general problems, their interrelationships, and constrictions.**

In this study, the three previously mentioned problems: routing, scheduling, and loading will be investigated together in parallel to minimize number of trains, inventory costs, and system variability as shown in the right side of Figure 1. The three problems and their interrelationships which will be explained later are shown in Figure 2. During working on these problems, four limitations, which are tugger train capacity, line-side inventory limit, time buffers, and routing time, as shown in the corners of Figure 2 are considered

To achieve the objectives, the general procedures of the study are shown in Figure 1 where we have 5 major steps. The first step was explained above in the example. The rest of the steps are explained in the next part of the study.

### Period length determination

In this study, it is assumed that routing is fixed, that is, if a tugger train feeds in the first route the first 5 stations for example, the same train will also feed the same five stations in every next route. It is also assumed in this study that the periods are the same during the shift, that is, the train feeds some certain stations every 30 minutes for example. This 30-minutes period remains the entire shift. Therefore, if the train makes its first route in just 20 minutes, it must wait another 10 minutes until it starts the next route. However, the period length for each train may be different from that for other trains.

To determine the period length, at first, the tugger train has a certain capacity that cannot be exceeded. Another restriction is the maximum allowed line-side inventory beside stations. These two restrictions push the period not to be too long. On the other hand, the period must account for the time needed for the train to move from the decentralized supermarket to the stations, from a station to the next one, and from the stations back to the supermarket including all the times of loading and unloading of empty and full bins. To find the period length for each group of stations supplied by the same train, at first the maximum possible period length for the train with a route (cell) from station *s*_*i*_ to station *s*_*j*_ including the station *s* is computed. To do that at first, the minimum number of routes is computed based on the maximum line-side inventory (MLSI) and the train capacity. Minimum Number of Routes for the cell (*s*_*i*_, *s*_*j*_), MNR (*s*_*i*_, *s*_*j*_), can be calculated based on Equation 11

Where the Minimum Number of Routes for Station *s*, *MNRS(s)*, which is computed based on MLSI, can be estimated using Equation (2)2

Where [*X*] is the lower rounded integer value for the variable *X*, and [*X*] is the upper rounded integer value for the variable *X*. The MLSI needed for a certain station is based on the maximum delivered number of bins at that station at any route. It is assumed in this study that any bin from which some parts were consumed by the station is not considered in the calculation of the line-side inventory.

The MAXimum Period Length, MAXPL (*s*_*i*_*, s*_*j*_) can be estimated using Equation (3)3

The MINimum Period Length, MINPL (*s*_*i*_*, s*_*j*_), is computed based on the time buffer, the Routing Time Inside the cell (*s*_*i*_*, s*_*j*_), RTI (*s*_*i*_*, s*_*j*_), and Routing Time Outside the Cell (RTOC) as in Equation (4)4

In the example above, RTI (*s*_*i*_*, s*_*j*_) is estimated to be *s*_*j*_*-s*_*i*_ +1 since the loading and unloading of bins are assumed to take 1 cycle for every station inside the cell. RTOC was assumed to be the same for all cells and equal to 2.

The feasible Period Length Range (PLR) can be computed using Equation (5)5

In the case that PLR (*s*_*i*_*, s*_*j*_) equals zero, the solution is not feasible. So based on the previous constrictions, there can be no cell starting from station *s*_*i*_ to station *s*_*j*_*.*

After finding the feasible space, the optimal period length for the cell (*s*_*i*_*, s*_*j*_) is set to be the minimum since it gives minimum inventory costs, and this coincides with the principle of JIT in which frequent small replenishments of materials are done. So the maximum period length shown above was only computed to find the feasible solution space.

As stated before, in this study, the scheduling problem consists of two parts: finding the value of period length, and finding the point of time at which the first movement of the train is started. So far, we did the first part, but the second part is interrelated to the loading problem, as will be shown later.

### Number of trains and new feasible solution space

**Figure 3:**
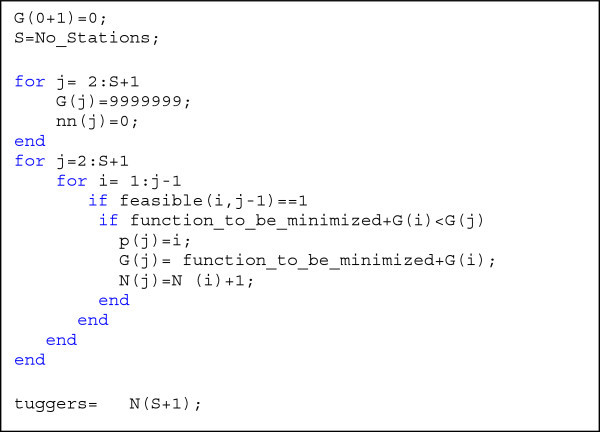
**DP in routing problem.**

In this study, we assume that the most important objective is minimizing the number of trains. So the first step is to find the minimum possible number of trains regardless of the other two objectives. Dynamic Programming (DP) is used to find this number. As shown in the study by (Emde and Boysen 2012a), DP in routing problem can be formulated as in Figure 3 which was programmed by Matlab software which does not accept zero indexing in which the index is zero. Therefore, 1 is added to zero. The idea of dynamic programming model is not to try all the possible combinations in the feasible solution space, and this is to save time. So, in an intermediate step, if we know the optimal solution (routing) for a group of stations (from 1 to *j*-1), then in a next step if we need to find the optimal routing for the same group of stations, there is no need to repeat the solution since the model “memorizes” the best solution found before. In this case, the time needed to find the final solution is minimized.

The variable *N* was added to find the number of tugger trains. Because we want the number of trains to be close to the minimum possible value which is one train, the function to be minimized here is set to represent the difference between the total number of stations (S) and the cell length. This function can be written as in Equation (6)6

Where, the value (*j-i*) represents the real cell length containing the stations from *i* to *j*-1.

Based on the found minimum number of trains, a new and smaller feasible solution space is found. To do that, DP will be used again for every possible cell based on the previous feasible space. For each feasible cell (*s*_*i*_*, s*_*j*_), DP is tried, where the function to be minimized now is (*total number of stations-*(*j-i*) *+ cost*(*i,j-*1)). *cost* matrix contains high values, for example 1000, except for *cost*(*s*_*i*_*, s*_*j*_), it contains very low value, for example 1. By this way, the cell (*s*_*i*_, *s*_*j*_) is very “attractive” to be chosen by the model in the optimal routing. However, there is another factor (*number of stations-(j-i))* which prohibits any cell that causes the number of trains to be more than the optimal one found before. For every point (*i, j-*1), the optimal solution is found and the active cells are determined in the solution. After doing that for all the points of the feasible solution space found before using Equation (5), all the active cells are put in the new feasible space. This step is done to minimize the computation time in the next step in which the final optimal solution is found.

**Figure 4:**
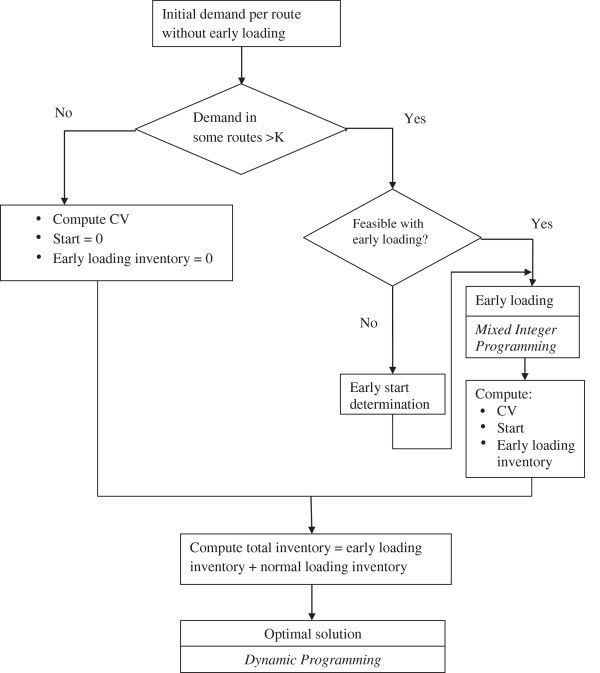
**General Framework for the last step in the study.**

Figure 4 represents the general framework of the last step found in Figure 1. In this step the other types of objectives are considered. At first, the Average Route Length (ARL) is computed by dividing the number of stations by the number of tuggers. We want cell lengths in the final solution not to be very far away from ARL. So this represents the first type of variability in the system. The second type is the variability in loading. The loaded quantity by each train should be almost the same during the whole shift. To measure that, the Coefficient of Variation (CV), of the quantity loaded by the train during the shift, is computed. This value is computed for all the feasible cells. However, if this variability in demand is so high to the level that in some routes the needed quantity is more than the capacity of the train, the loaded quantity does not need to be equal to the demand all the time. So we must do *early loading* for some of the needed bins in previous non-bottleneck routes before these bins are needed. However, this will increase the inventory holding costs. In this study, a simple rule is followed: if increasing one type of costs will decrease two types of costs, we should do it, as shown in the lower right part of Figure 1. *Early loading* of bins will increase the inventory holding costs, but it will give us the chance to decrease the number of trains and also to decrease the variability in the system.

However sometimes even early loading is not enough to minimize the needed capacity of trains. This can happen if the first route demand is more than the capacity of the train. Therefore, the train can adopt *early start*. So, early start is needed to define the time of the beginning of the first route. Until now, that time was one cycle before the demand of stations. For example, if the first station needs parts at cycle number one, the needed parts must be delivered one cycle before (cycle number zero). In early start and to decrease the demand of the first route, we add the possibility for the train to start its movement several cycles before the first time the parts are needed. The demand of the stations in every route including the first one will be changed. The first route will cover a period that is equal to usual period length minus the early start period. Another factor is that the decision maker may not want the number of routes to be increased. In this case, the last route will cover a period that is longer than the usual periods before. The methodology to find the early start is trying different early start periods from zero to the first feasible one. The feasibility is checked by constriction (7). If it is true for all routes, then feasibility is achieved. After finding the start point, the loading problem is investigated.7

Where,

T Total number of routes

S Number of stations

K Capacity of the train

*d*_*st*_ Demand bins of the station *s* for the route *t*

### Loading problem

Loading problem was formulated by (Emde et al. 2012). The constraints were as follows8910

And there were two objectives as follows:1112

Where, *x*_*st*_ is the delivered number of bins at station *s* in the route *t*.

Constraint (8) guarantees that the capacity of the tow train will not be exceeded. Constraint (9) guarantees that for every route, the accumulated number of delivered bins to a station is at least equal to the accumulated demands by this station until the current period (route). Constraint (10) defines the positive integer number of bins that can be delivered to different stations. The objective function (11) aims at minimizing the difference between the needed demand and delivered number of bins. Furthermore, the maximum amount of bins stashed at any one station should be minimal, therefore, there is another objective (12) which minimizes the maximum difference between the numbers of delivered bins and the demand of the bins. Emde et al. (2012) used a new heuristic to solve the problem to find the value of *f*_*max*_. However, there is another simpler but slower way to solve it by adding a new constraint (13) instead of the second objective, and also by adding the variable *z* to the first objective.13

The results of the new model is exactly as that found by the heuristic defined in Emde et al. (2012) since the value of *z* equals *f*_*max*_. To formulate all the problems together in the same program, Matlab software in conjunction with LP-solve software was used.

Further investigation in this loading model can be considered. For example constriction (14) can be added14

The variable *v* is added to the objective function. This constraint is to minimize the maximum difference between the train capacity and the loaded quantity. This will decrease the variability in the loading since it pushes the lowest loaded quantity to be higher. However, this can increase inventory costs since it increases the early loaded quantity.

Another important factor is MLSI. For example, Tables 3a and b shows the data of an example presented in the study by Emde et al. (2012) and was used for explaining loading problem where the maximum delivered number of bins, 13, was for the 4^th^ station in the 3^rd^ route. There is, however, another alternative optima with the same objective function value but with lower maximum loaded quantity, 11, in Table 3c. So if MLSI is only 11 units, the second solution is the feasible one. As a matter of fact, there are a lot of alternative optima for this example. To take that into consideration, another constraint can be added as in constriction (15)15

**Table 3:** **Alternative optima for loading problem**

***d***_***st***_	1	2	3	4	5	***x***_***st***_	1	2	3	4	5	***x***_***st***_	1	2	3	4	5
1	0	7	0	8	0	1	4	7	0	4	0	1	4	6	1	4	0
2	0	7	0	8	10	2	4	7	0	8	6	2	4	6	1	8	6
3	6	0	10	3	10	3	6	4	7	6	6	3	6	4	7	6	6
4	6	0	15	0	10	4	6	2	13	2	8	4	6	4	11	2	8
a. Demand per station and route						b. Optimal solution						c. Alternative optima					

Another dimension that can be added to the model is minimizing the frequency of *f*_*max*_. For example in Table 3b and c even if the objective function values are the same, the frequency of *f*_*max*_ is different. Of course, the less times *f*_*max*_ appears, the better the results are. This can be done by adding two constraints (16) and (17)1617

The value *y2*_*st*_ equals 1 if *f*_*max*_ appears at station *s* and in the route *t.* So the value  must then be added to the objective function. For this objective not to affect the original objectives defined by Emde et al. (2012), this value must have a very small weight compared to the two original objectives.

The decision maker may want to further investigate the *f*_*max*_ value by reducing the maximum number the *f*_*max*_ values appear for the same station at successive routes by adding the objective (18)18

This can be accomplished by adding a new constraint (19) and by adding the variable *length* in the objective function.19

However, this will make the problem too complicated and make it difficult to solve. Another factor that the decision maker may consider is the number of stops of the tugger train. For example in the solution in Table 3b the number of zeros was three but in the other solution in Table 3c the number of zeros is just one. The more zeros, the less number of stops of the train. This may be useful to reduce the “traffic jam” of tugger trains especially in the case of using narrow aisles in the facility. This can be done by adding a new constraint (20) and adding the value  in the objective function. However, this objective contradicts the objective of minimizing the maximum line-side inventory. The decision maker can decide if he wants to add such an objective or not according to the situation on the ground.20

### Optimal solution

DP is used one more time to find the final solution. The function to be minimized contains all the types of costs except the number of trains since it was already considered before in finding the second feasible space. So the function to be minimized for the cell from station *i* to station *j-*1 is as in Equation (21)21

Where *w*_*i*_ is the weight of objective *i*, and *total_INV_costs* represents the normal loading inventory holding costs plus early loading inventory holding costs. The values of the weights can be estimated according to the judgment of the decision maker. Because the early loading inventory is in the system for routes time but the normal loading inventory is in the system only for cycles time, the last one is divided by period length to transform it to routes time unit. At first, it is computed by multiplying the amount of needed inventory in each cycle and for each station by the time period from that cycle to the time of delivering that inventory beside the station, and this is the same way followed by Emde et al. (2012). So in Table 2b and assuming that the first 5 stations form a cell with period length of 7, and we want to compute the holding costs for normal loading for the third station and for only the first route, the train comes and unloads two bins in the second cycle. In the third cycle, station 3 will start consuming the first bin immediately so its inventory cost is zero. The inventory cost for the second bin is 5 since it will wait 5 cycles until it is started to be consumed. The early loading inventory is computed based on the objective function value introduced in (11) and (12). The method above about computing normal loading inventory cost is also applied for bins that are consumed in the current route but delivered in previous routes since these bins will also wait for few cycles in the current route beside, of course, the waiting from route to route.

### Interrelations among problems

As it is obvious in Figure 2, to do routing problem, feasible range of period length must be defined first, and this is part of scheduling problem. Moreover, to define early start which is part of scheduling problem, loading problem must be considered. However, loading problem cannot be done without knowing the period lengths found in scheduling problem. Furthermore, the optimal routing needs information about the values of the objective function from loading problem (inventory costs and CV), and also the minimum possible number of trains which must be found based on the feasible range found in scheduling problem. All that reveals the importance of investigating the three problems together in parallel.

## Results and analysis

At first, the weight of the objective function in Equation (21) was set to be 100 for the *CV* value since it is usually very small and we want for this value to affect the solution. Each one in the other two parts in the objective function was given a weight of 1. The time buffer among routes was set to be 1 cycle. All the problems were run together and needed just few seconds on a normal personal computer.

The results of the example are in Table 4 where the feasible space is in Table 4a. The value of 1 is for the active cells in the feasible space. For example the final solution must have a cell containing the first 5, 6 or 7 stations. In the optimal solution, there are three trains, and one of them supplies the stations from 1 to 7, the second one supplies the stations from 8 to 14, and the last one supplies the rest of the stations. The scheduling results are shown in Table 4b expressed in the form (*x, y*) where *x* is the period length and *y* is the early start. There was no early loading at all in the optimal solution. We will, however, have early loading in the third cell if the weight of CV value is increased to be 102, and the results will contain three cells, where the first one contains the stations from 1 to 6, the second cell contains the stations from 7 to 13, and the third cell contains the remaining stations. These changes in results show the effect of the objective, which decreases the variability in loading, on the routing, scheduling, and loading results. The effect on routing is not in the number of trains but in the cells formation.

**Table 4:** **Feasible space and optimal solution when time buffer is 1 cycle**

Stations	5	6	7	13	14	20	Stations	7	14	20
1	1	1	1				1	(10, 0)		
6				1			6			
7				1			7			
8				1	1		8		(10, 0)	
14						1	14			
15						1	15			(9, 0)
a. Feasible space							b. Optimal solution			

Table 5 shows the results when the time buffer is set to be zero where the value of the objective function is decreased from 487.3 to 452.1 and the feasible space is increased to contain more cells. Furthermore, the last cell from station 13 to 20 has early loading and also early start of 1 cycle. The first route is too short compared to the other two ones. To fix this problem, the weight *w*_1_ in Equation (21) must be increased. If it is set to be 21 for example, the new solution will contain a cell from station 1 to 6, another cell contains stations from 7 to 12, and the last one contains the remaining stations. However, if this weight is increased to be 67, the first cell will be from station 1 to 7, the second cell will be from station 8 to 13, and the last cell will contain the remaining stations.

**Table 5:** **Feasible space and optimal solution when time buffer is 0**

Stations	4	5	6	7	8	12	13	14	15	16	20	Stations	4	12	20
1	1	1	1	1	1							1	(6, 0)		
5						1						5		(10, 0)	
6						1	1					6			
7						1	1					7			
8						1	1	1				8			
9						1	1	1	1	1		9			
13											1	13			(10, -1)
14											1	14			
15											1	15			
16											1	16			
17											1	17			
a. Feasible space												b. Optimal solution			

Table 6 shows several trials in which the K and MLSI values were changed to get the optimal objective function value and the number of needed trains. The 4^th^ trial represents the current status. If we decrease the K value to be 10, we will need 4 trains. It is noted that no matter how much we increase the K value more than 16, it cannot affect the results. This is because of the fixed value of MLSI. So to get lower number of trains, the MLSI value must be increased as in the 10^th^ trial. This result is important since it shows that it is not very helpful to get tow trains with high capacities if the maximum line-side inventory cannot be increased.

**Table 6:** **Effect of K and MLSI values on the results**

Trial	K	MLSI	Number of trains	O.F. value
1	17	3	3	474.96
2	16	3	3	474.96
3	15	3	3	452.12
4	14	3	3	452.12
5	13	3	3	526.17
6	12	3	3	592.30
7	11	3	3	600.93
8	10	3	4	548.33
9	14	4	3	452.12
10	21	4	2	630.67

## Conclusion

In this study the routing, scheduling, and loading problems of the tow train were investigated together in parallel to decrease the number of trains, variability in loading and in route lengths, and inventory holding costs in normal and in early loading. This was done using analytical equations, DP, and MIP techniques. Constrictions related to tugger capacity, line-side inventory maximum limit, routes time, and time buffer among routes were taken into consideration. Beside the time buffer, safety stock and empty space capacity for line-side inventory can be used. Further investigation about loading problem was presented using MIP.

This study presents a systematic way to manage material flow using milk run trains containing the three problems of routing, scheduling, and loading. It also shows the importance of studying the three problems together since they are interrelated, and to minimize the total inventory holding costs to the minimum possible value and to take into account decreasing the variability in the loaded quantities. It also shows the effect of time buffer on the feasible solution space. Moreover, it shows the importance of considering the maximum line-side inventory and the capacity of the train in the same time, where getting tugger trains with so high capacities does not enhance the system if the line-side inventory is still limited to small amounts. It also shows the importance of the objective regarding decreasing the variability in loaded quantities since it affects all the results of routing, scheduling, and loading.

In this study there are some limitations. At first, the study assumes that the exact number of product models and their sequence is known before the beginning of the shift. Moreover, the study is applicable only if the management decided not to use kanban system. Furthermore, the supermarket system is assumed. This means that the study is not applicable if central warehouse is used instead of supermarket system.

In future research, a comparison between kanban system and the system in this study can be made to investigate when it is advisable to use kanban system. Also, it is recommended in future research to investigate such problems in real case studies.

## References

[CR1] Alizon F, Dallery Y, Essafi I, Feillet D (2009). Optimizing material handling costs in an assembly workshop. Int J Prod Res.

[CR2] Álvarez R, Calvo R, Peña M, Domingo R (2009). Redesigning an assembly line through lean manufacturing tools. Int J Adv Manuf Technol.

[CR3] Battini D, Faccio M, Persona A, Sgarbossa F (2009). Design of the optimal feeding policy in an assembly system. Int J Prod Econ.

[CR4] Battini D, Faccio M, Persona A, Sgarbossa F (2010). “Supermarket warehouses”: stocking policies optimization in an assembly-to-order environment. Int J Adv Manuf Technol.

[CR5] Baudin M (2004). Lean logistics: the nuts and bolts of delivering materials and goods.

[CR6] Bozer Y, Ciemnoczolowski D (2013). Performance evaluation of small-batch container delivery systems used in lean manufacturing – Part 1: system stability and distribution of container starts. Int J Prod Res.

[CR7] Caputo AC, Pelagagge PM (2011). A methodology for selecting assembly systems feeding policy. Ind Manage Data Syst.

[CR8] Choi W, Lee Y (2002). A dynamic part-feeding system for an automotive assembly line. Comput Ind Eng.

[CR9] Ciemnoczolowski D, Bozer Y (2013). Performance evaluation of small-batch container delivery systems used in lean manufacturing – Part 2: number of Kanban and workstation starvation. Int J Prod Res.

[CR10] Domingo R, Alvarez R, Peña M, Calvo R (2007). Materials flow improvement in a lean assembly line: a case study. Assem Autom.

[CR11] Droste M, Deuse J (2011). A planning approach for in-plant milk Run processes to optimize material provision in assembly systems.

[CR12] Emde S, Boysen N (2012). Optimally routing and scheduling tow trains for JIT supply of mixed-model assembly lines. Eur J Oper Res.

[CR13] Emde S, Boysen N (2012). Optimally locating in-house logistics areas to facilitate JIT-supply of mixed-model assembly lines. Int J Prod Econ.

[CR14] Emde S, Fliedner M, Boysen N (2012). Optimally loading tow trains for JIT-supply of mixed-model assembly lines. IIE Trans.

[CR15] Faccio M, Gamberi M, Persona A, Regattieri A, Sgarbossa F (2013). Design and simulation of assembly line feeding systems in the automotive sector using supermarket, kanbans and tow trains: a general framework. J Manag Control.

[CR16] Golz J, Gujjula R, Günther H-O, Rinderer S, Ziegler M (2011). Part feeding at high-variant mixed-model assembly lines. Flex Serv Manuf J.

[CR17] Hanson R, Finnsgård C (2012). Impact of unit load size on in-plant materials supply efficiency. Int J Prod Econ.

[CR18] Kilic HS, Durmusoglum MB, Baskak M (2012). Classification and modeling for in-plant milk-run distribution systems. Int J of Advanced Manufactory Tech.

[CR19] Sargent T, Michael G, Kay M (1995). Implementation and utilization of a decentralized storage system: costing model. Int J of Operations & Prod Mngt.

[CR20] Satoglu S, Sahin E (2012). Design of a just-in-time periodic material supply system for the assembly lines and an application in electronics industry. Int J of Adv Manufactory Tech.

